# Efficacy of a Laparoscopic Approach in Treating Gangrenous Ischemic Colitis: A Case Report

**DOI:** 10.70352/scrj.cr.25-0745

**Published:** 2026-04-10

**Authors:** Megumi Sugai, Hideaki Kojima, Ippei Onishi, Tomonori Fujimura, Hiroaki Yamada, Wenlin Du, Hitoshi Satodate

**Affiliations:** 1 Department of Surgery, Kawasaki Municipal Ida Hospital, Kawasaki, Kanagawa, Japan; 2 Department of Gastroenterology, Kawasaki Municipal Ida Hospital, Kawasaki, Kanagawa, Japan; 3 Department of Pathology, Kawasaki Municipal Ida Hospital, Kawasaki, Kanagawa, Japan

**Keywords:** ischemic colitis, gangrenous, emergency surgery, laparoscopic surgery

## Abstract

**INTRODUCTION:**

Ischemic colitis (IC) is one of the most common ischemic disorders of the gastrointestinal tract. Although most cases can be managed conservatively, some progress to the gangrenous form requiring surgical intervention. Emergency surgery for gangrenous IC has typically been performed via laparotomy.

**CASE PRESENTATION:**

An 81-year-old woman presented with abdominal pain and hematochezia. She was initially managed conservatively under the diagnosis of IC; however, on hospital day 4, she developed peritoneal signs and a marked increase in inflammatory markers. Contrast-enhanced CT revealed poor enhancement of the splenic flexure and descending colon. Diagnostic laparoscopy confirmed gangrenous IC, and a laparoscopic extended left hemicolectomy with colostomy was performed. The postoperative course was uneventful, and the patient achieved a favorable outcome.

**CONCLUSIONS:**

This case highlights that laparoscopic surgery is a safe and effective option for diagnosing and managing gangrenous IC in selected patients.

## Abbreviations

CRP
: C-reactive protein
H&E
: hematoxylin and eosin
IC
: ischemic colitis
SD
: sigmoid descending
WBC
: white blood cell

## INTRODUCTION

IC, the most common ischemic disorder of the gastrointestinal tract, predominantly affects older women.^1)^ It accounts for 16%–24% of patients presenting with gastrointestinal bleeding,^2)^ with an estimated incidence of 4.5–44 cases per 100000 person-years in the general population.^3–5)^ IC is caused by transient, nonocclusive ischemia localized to the colon. Although its precise pathogenesis remains unclear, microvascular disease has been implicated, and risk factors such as hypertension, hyperlipidemia, diabetes mellitus, coronary artery disease, and arrhythmia have been reported.^6)^ Clinically, IC typically presents with an acute onset of lower abdominal pain, diarrhea, and hematochezia. Most cases are transient and resolve with supportive care; however, a subset progresses to the gangrenous form, which accounts for approximately 12.8% of cases and requires surgical intervention.^7,8)^

Emergency surgery for gangrenous IC has typically been performed via laparotomy, and laparoscopic approaches remain uncommon. According to the American College of Surgeons National Surgical Quality Improvement Program, laparoscopic colectomy was performed in only 125 of 4548 patients (4.3%) with IC.^9)^ Although the usefulness of laparoscopic surgery has been increasingly reported for various emergency abdominal conditions, existing studies on acute colonic diseases have mainly focused on perforated diverticulitis, anastomotic leakage, or inflammatory colitis,^10–13)^ and evidence regarding laparoscopic surgery for gangrenous IC remains limited. Gangrenous IC is a diagnostically challenging condition, and delays in therapeutic intervention are common due to its often nonspecific clinical presentation and the difficulty of definitive preoperative diagnosis. In this context, the application of laparoscopic surgery may enable a stepwise approach, allowing accurate diagnosis through direct intra-abdominal assessment followed by timely definitive treatment in patients with suspected gangrenous IC. Herein, we report a case of gangrenous IC that was successfully treated with emergency laparoscopic surgery, highlighting the potential utility of a staged laparoscopic approach.

## CASE PRESENTATION

### Clinical presentation

**Fig. 1:**
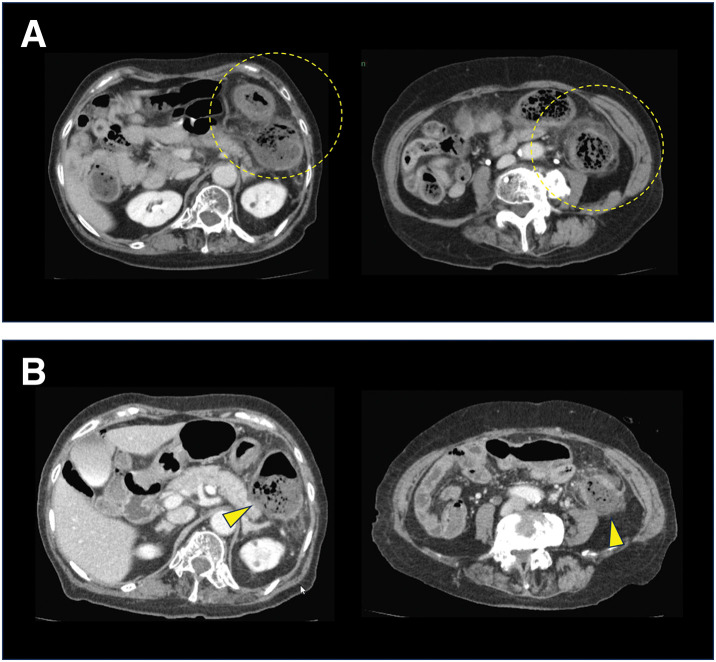
Contrast-enhanced CT scan images of the patient. (**A**) Contrast-enhanced CT scan images taken at the time of diagnosis of ischemic colitis. Edematous wall thickening and the dirty fat sign are observed in the splenic flexure and the descending colon (yellow circle). (**B**) Contrast-enhanced CT images taken at the time of progression to gangrenous ischemic colitis. Contrast enhancement defects and thinning of the colonic wall are observed in the splenic flexure and descending colon (yellow arrowhead).

An 81-year-old woman presented to the emergency department with the sudden onset of intermittent left upper abdominal pain and hematochezia. She had a history of hypertension but no other significant comorbidities and was not taking any regular medications. She was afebrile and her vital signs were stable. Physical examination revealed a soft, flat abdomen with localized tenderness in the left flank, but no signs of peritoneal irritation. Laboratory tests showed a hemoglobin level of 13.4 g/dL, a WBC count of 9200/µL, and a CRP level of 0.09 mg/dL, indicating no anemia or inflammatory response. Contrast-enhanced abdominal CT demonstrated colonic wall thickening with increased pericolic fat density (dirty fat sign) around the splenic flexure, without evidence of mesenteric vessel occlusion (**Fig. 1A**). She was admitted with a diagnosis of IC.

On hospital day 2, although her abdominal symptoms did not worsen, she developed a low-grade fever of 37.6°C. Laboratory findings revealed a WBC count of 7200/µL and a CRP level of 5.65 mg/dL, indicating only a mild inflammatory response. On hospital day 4, her abdominal pain worsened, and signs of peritoneal irritation appeared. Blood tests revealed marked elevations of her WBC count (10400/µL) and CRP level (37.32 mg/dL). Repeat contrast-enhanced CT showed localized areas of poor enhancement in the colonic wall at the splenic flexure and descending colon, with no evidence of perforation (**Fig. 1B**). These findings raised concern for the progression to gangrenous IC, and emergency surgery was planned.

### Surgical strategy

The initial surgical strategy was to perform diagnostic laparoscopy to confirm the presence of colonic necrosis and to assess the extent of the disease. Colectomy was planned if serosal necrosis was identified intraoperatively.

This patient had no history of abdominal surgery, was hemodynamically stable, and contrast-enhanced CT demonstrated a localized lesion without perforation or significant intra-abdominal contamination. In addition, she was not obese (height 144 cm, weight 39.6 kg, BMI 19.0). Accordingly, no factors that would typically hinder a laparoscopic approach were present. Based on these considerations, laparoscopic colectomy was planned as the primary surgical approach following diagnostic laparoscopy. Primary anastomosis was not planned because of the increased risk of anastomotic leakage, given the patient’s advanced age, the emergency setting, and the ischemic etiology of the disease.

### Surgical procedure

**Fig. 2:**
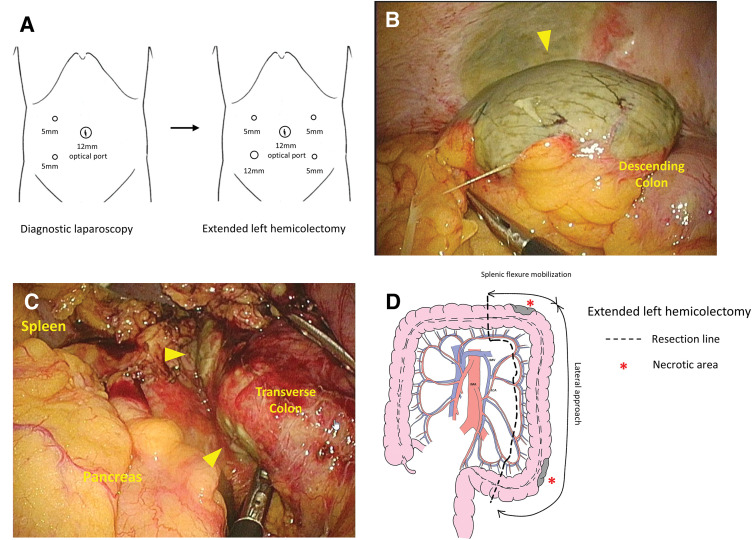
Intraoperative findings of the presented case. (**A**) Schematic diagram of port placement for diagnostic laparoscopy and colectomy. (**B**) Appearance of the descending colon. A partial full-thickness ischemic necrosis and thinning of the colonic wall are observed (yellow arrowhead). (**C**) Appearance of the splenic flexure of the colon. Marked inflammation of the transverse colon and partial full-thickness ischemic necrosis (yellow arrowheads) is observed. (**D**) Schematic diagram of the extent of intestinal and mesenteric resection.

**Fig. 3:**
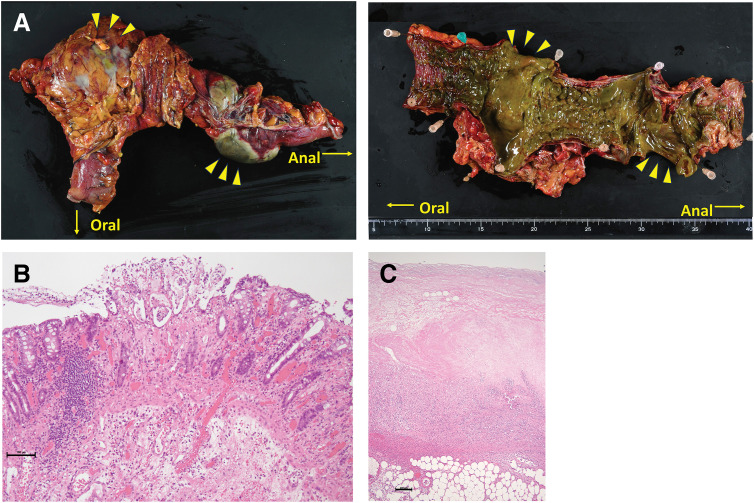
Pathological findings in the resected specimen. (**A**) Macroscopic appearance of the resected left-sided colon. The length of the resected segment was 33.0 cm. Areas of full-thickness ischemic necrosis are observed (yellow arrowheads). (**B**) H&E staining image of the edematous thickening part of the resected colon. Prominent inflammatory cell infiltration, submucosal edema, and congestion are observed. Scale bar = 100 µm. (**C**) H&E staining image of the necrotic area of the resected colon. Full-thickness ischemic coagulative necrosis is observed. Scale bar = 200 µm. H&E, hematoxylin and eosin

The operation was performed by an experienced surgical team specializing in gastrointestinal surgery, with supervision by a board-certified endoscopic surgeon accredited by the Japan Society for Endoscopic Surgery. The patient was placed in the lithotomy position, and ports were placed as shown in **Fig. 2A**. Pneumoperitoneum was established at 10 mmHg. Laparoscopic exploration revealed inflammatory adhesion of the greater omentum to the colon near the SD junction. After careful adhesiolysis, a localized segment of greenish, thinned colonic wall consistent with necrosis was identified (**Fig. 2B**). Partial opening of the omental bursa allowed inspection of the distal transverse colon, which revealed an additional area of segmental necrosis consistent with the preoperative CT findings, indicating skip lesions (**Fig. 2C**). No serosal color changes were observed in the ascending colon, proximal transverse colon, or rectum. Based on these findings, the diseased segment was determined to extend from the distal transverse colon to the SD junction. Although inflammatory changes were present, the remaining colon and mesocolon were amenable to gentle traction, and laparoscopic colectomy was deemed technically feasible. Additional ports were placed, and laparoscopic colectomy was initiated. Using a lateral approach, inflammatory adhesions and physiological fusion between the SD junction of the colon and the left abdominal wall were carefully dissected. Gentle traction was applied to avoid tearing the thinned colonic wall, and mobilization was performed from the lateral aspect of the rectum to the splenic flexure. Subsequently, the attachment between the greater omentum and the transverse colon was divided toward the splenic side and connected to the lateral mobilization. The transverse mesocolon was then detached at the inferior border of the pancreas, allowing complete mobilization of the distal transverse colon. Given the patient’s slender body habitus, the colon could be adequately exteriorized through the umbilical incision. As lymphadenectomy was not required, the mesentery and associated vessels were divided extracorporeally along the bowel, and the colon was transected using a linear stapler (**Fig. 2D**). Considering the potential for proximal or distal progression of ischemia and the risk of stump rupture associated with a long residual rectum, the transection lines were placed at a sufficient distance from the necrotic bowel, in areas with minimal inflammatory changes on preoperative CT. Although superficial mucosal erosion was observed at the stoma site and was more extensive than the serosal changes, adequate blood flow at the colonic stump was confirmed, and no additional resection was performed. A laparoscopic extended left hemicolectomy without primary anastomosis was completed, and a transverse colostomy was constructed. A drainage tube was placed along the Douglas pouch and the left lateral wall. The operative time was 2 h 59 min, and the estimated blood loss, including purulent ascites, was 100 mL. Macroscopic examination of the resected specimen showed greenish necrotic mucosa and heterogeneous edematous thickening and thinning of the colonic wall (**Fig. 3A**). Histopathological examination demonstrated mucosal erosion, edema, and transmural congestion consistent with IC (**Fig. 3B**), with full-thickness ischemic coagulative necrosis in the thinned areas (**Fig. 3C**). The final diagnosis was acute gangrenous IC.

The postoperative course was uneventful. The patient passed flatus on POD 2, and bowel function recovery was confirmed by defecation on POD 3, at which point oral intake was started. No ischemic changes of the stoma were observed throughout the clinical course. She was discharged without complications on POD 16.

## DISCUSSION

Surgical resection of the affected bowel remains the only definitive treatment for gangrenous IC, and conservative management in such cases is potentially fatal. In the present case, the patient had no apparent risk factors other than hypertension and advanced age, and the initial clinical presentation and laboratory findings were consistent with typical, nonsevere IC, generally amenable to conservative treatment. The absence of hemodynamic instability, including paroxysmal arrhythmia, further obscured early recognition of disease progression. In clinical situations where the need for bowel resection is difficult to determine, a stepwise laparoscopic approach that integrates both diagnosis and treatment may offer several advantages. To date, the available evidence regarding laparoscopic surgery for gangrenous IC has been limited largely to postoperative outcomes, with little emphasis on its diagnostic and surgical utility. In this report, we demonstrate the practical value of this stepwise diagnostic and therapeutic strategy and provide clinically applicable insights for physicians and surgeons involved in the care of patients with acute IC.

IC may occur throughout the colon, and in our case, transmural necrosis was identified at two separate sites: the splenic flexure and the descending colon. Given the possibility of skip lesions, the ability to examine the entire colon through minimal incisions is a significant advantage of a laparoscopic approach. Anatomically, the splenic flexure, located at the watershed area between the superior and inferior mesenteric circulations, is particularly susceptible to ischemia.^14)^ However, this site is difficult to visualize during laparotomy, often requiring extended incisions. In contrast, laparoscopic surgery allows adequate resection with minimal invasiveness, even when colectomy becomes necessary. Another clinical challenge is that IC is usually self-limiting and managed conservatively, which raises the threshold for surgical intervention. Even when gangrene is suspected, endoscopic evaluation is often avoided due to concerns about perforation. Reportedly, endoscopic findings influenced surgical decision-making in only 10% of IC cases, with a high discordance rate (50%) between mucosal and serosal findings.^15)^ On the other hand, the mortality of patients with gangrenous IC who require surgery is extremely high, reportedly over 50%,^16)^ underscoring the importance of early diagnosis. A surgical delay exceeding 12 h is an independent predictor of postoperative mortality,^17)^ whereas early colectomy before the onset of shock associated with colonic necrosis reportedly reduces mortality.^18)^ In this context, diagnostic laparoscopy, as a minimally invasive and readily acceptable step, provides a valuable advantage.

Determining the appropriate extent of bowel resection is another important issue and is complicated by several factors: (i) in nonocclusive IC, as in the present case, mesenteric and marginal arterial anatomy does not reliably indicate the resection margin; (ii) the disease may progress over time; and (iii) mucosal lesions often extend more widely than serosal changes,^15)^ leading to discrepancies in intraoperative assessments. In this case, the extent of bowel resection was determined by integrating intraoperative serosal findings with preoperative contrast-enhanced CT imaging. Specifically, the areas suspected of necrosis on CT corresponded precisely to the segments showing transmural necrosis on intraoperative inspection. These concordant findings established the necessity of resecting the affected segments, and the bowel was transected with an adequate margin at sites where inflammatory changes were minimal on preoperative imaging. Nevertheless, given the potential for progression of ischemia beyond the macroscopically affected bowel, alternative strategies—such as the creation of a distal mucous fistula—may warrant consideration in selected cases. In addition, intraoperative colonoscopy or near-infrared indocyanine green angiography may provide further support in delineating appropriate resection margins by enabling more precise assessment of mucosal viability and regional perfusion.

In recent years, laparoscopic surgery has been increasingly applied to patients with inflammatory diseases and emergency abdominal conditions, with favorable outcomes reported.^19)^ Although evidence regarding laparoscopic surgery for IC remains limited, some studies have demonstrated its feasibility and safety, with reduced postoperative surgical site complications and a shorter duration of ventilatory support compared with open surgery.^17)^

Based on the characteristics observed in the present case, laparoscopic surgery for gangrenous IC may be considered in carefully selected patients who meet the following clinical conditions: (i) hemodynamic stability; (ii) absence of diffuse fecal peritonitis; (iii) localized disease in which mobilization of the colon is technically feasible; (iv) absence of factors that would compromise laparoscopic visualization, such as severe obesity, multiple prior abdominal surgeries, or extensive paralytic ileus associated with severe inflammation; and (v) suspected skip lesions requiring thorough intra-abdominal inspection.

However, even when these criteria are met preoperatively, prompt conversion to open surgery should be considered intraoperatively if safe laparoscopic dissection cannot be maintained. Situations warranting conversion include severe inflammatory adhesions or marked tissue friability that hinder colonic mobilization, uncontrolled bleeding or organ injury, and inability to secure an adequate operative field.

Thus, although the indications for laparoscopic surgery in gangrenous IC remain limited, diagnostic laparoscopy can provide valuable information regarding the extent of disease and bowel viability, thereby facilitating appropriate intraoperative decision-making in carefully selected patients.

## CONCLUSIONS

In summary, this case demonstrates that a laparoscopic approach can be a feasible, safe, and diagnostically valuable option for selected patients with gangrenous IC. Given its ability to facilitate early diagnosis, assess disease extent, and enable minimally invasive colectomy, laparoscopy should be considered as a potential strategy in the management of this life-threatening condition.

## DECLARATIONS

### Availability of data and materials

The data of the current study are available from the corresponding author upon reasonable request.
